# Epidemiology of malaria in a village in the Rufiji River Delta, Tanzania: declining transmission over 25 years revealed by different parasitological metrics

**DOI:** 10.1186/1475-2875-13-459

**Published:** 2014-11-26

**Authors:** Anna Färnert, Victor Yman, Manijeh Vafa Homann, Grace Wandell, Leah Mhoja, Marita Johansson, Salome Jesaja, Johanna Sandlund, Kazuyuki Tanabe, Ulf Hammar, Matteo Bottai, Zulfiqarali G Premji, Anders Björkman, Ingegerd Rooth

**Affiliations:** Infectious Diseases Unit, Department of Medicine Solna, Karolinska Institutet, Stockholm, Sweden; Nyamisati Malaria Research, Rufiji, Tanzania; Osaka Institute of Technology, Osaka, Japan; Unit of Biostatistics, Department of Environmental Medicine, Karolinska Institutet, Stockholm, Sweden; Department of Parasitology and Entomology, Muhimbili University College of Health Science, Dar es Salaam, Tanzania; Department of Pathology, Aga Khan University Hospital, Nairobi, Kenya; Department of Microbiology and Tumour Biology, Karolinska Institutet, Stockholm, Sweden

**Keywords:** Malaria, Parasite prevalence, PCR, Microscopy, Spleen, Epidemiology, Transmission, Tanzania

## Abstract

**Background:**

Assessments of the epidemiology of malaria over time are needed to understand changes in transmission and guide control and elimination strategies.

**Methods:**

A longitudinal population study was established in 1985 in Nyamisati village in the Rufiji River Delta, Tanzania. A physician and research team lived in the village 1984–2000. Parasite prevalence by microscopy and two PCR methods, spleen rates and haemoglobin levels were measured in repeated cross-sectional surveys between 1985 and 2010. Passive surveillance of malaria cases was maintained until end 1999. Bed nets were distributed after the surveys 1993, 1999 and 2010.

**Results:**

In 1985, overall parasite prevalence by microscopy was 70% (90% in children ages two to nine years). The prevalence decreased gradually by microscopy (38.9% 1994, 26.7% 1999) and *msp2*-PCR (58.7% 1994, 44.8% 1999), whereas real-time PCR prevalence remained higher throughout the 1990s (69.4% 1994, 64.8% 1999). In 2010, parasite prevalence was 17.8% by real-time PCR and 16.3% by *msp2*-PCR, and estimated to 4.8% by microscopy. Spleen rates in children ages two to nine years decreased earlier than parasite prevalence, from >75 to 42% in the 1980s, to nil during the 1990s. The prevalence of severe and moderate anaemia decreased from 41.1 to 13.1%. No deaths at the time of acute malaria were recorded when the research team lived in the village.

**Conclusions:**

A marked decline in malaria transmission was observed over 25 years. The decrease was detected after the arrival of the research team and continued gradually both before and after distribution of bed nets. Spleen rates and microscopy identified early changes when transmission was still intense, whereas real-time PCR was a more sensitive metric when transmission was reduced. The study provides historical data on malaria within a closely monitored rural village and contributes to the understanding of changing epidemiology in sub-Saharan Africa.

**Electronic supplementary material:**

The online version of this article (doi:10.1186/1475-2875-13-459) contains supplementary material, which is available to authorized users.

## Background

Malaria remains a major global health problem. The infection is spread in tropical and subtropical regions with the most intense transmission in sub-Saharan Africa. Several countries have reported reduced transmission over the last decade [1]. Understanding the temporal changes in malaria transmission, determining factors that have contributed to these changes, and identifying optimal monitoring tools are important in designing future control interventions [2].

The Nyamisati Malaria Research Project was set up in 1985 in a fishing village in Rufiji District, Tanzania. A medical team established a health clinic in this community, which previously lacked primary health care, and became interested in understanding the impact of malaria. A research project, in which all villagers were invited, aimed to describe the epidemiology and clinical aspects of malaria longitudinally. The first cross-sectional surveys in 1985–1988 revealed a holo-endemic setting with 90% parasite prevalence and >75% spleen rates in two to nine years old children [3]. Repeated cross-sectional surveys were performed and a passive surveillance system for malaria was maintained until year 2000 when the team moved. Studies conducted within the project have described various aspects of malaria, e.g., epidemiology (1985–1988) and clinical presentation [3–5]; impact of co-infections with measles, influenza and pertussis [6]; molecular epidemiology and dynamics of *Plasmodium falciparum* populations [7–10]; parasite genetics and evolution [11, 12]; as well as immunology [13–16], and human immunogenetics [17, 18]. Reports of decreasing transmission in other African settings [19–21] inspired the research team to return to the village in 2010 to perform a cross-sectional survey to assess parasite prevalence.

This paper summarizes the epidemiology of malaria in Nyamisati village over 25 years. Long-term changes in *P. falciparum* prevalence were evaluated using microscopy and two PCR methods: species-specific real-time PCR and merozoite surface protein-2 (*msp2*) genotyping PCR. Moreover, spleen rates, haemoglobin levels and incidence of febrile malaria were assessed over time. The study shows a marked yet gradual decline in malaria transmission in Nyamisati, and demonstrates that different methods to monitor malaria endemicity are informative at different stages of changing transmission intensity.

## Methods

### Study area and population

**Figure 1:**
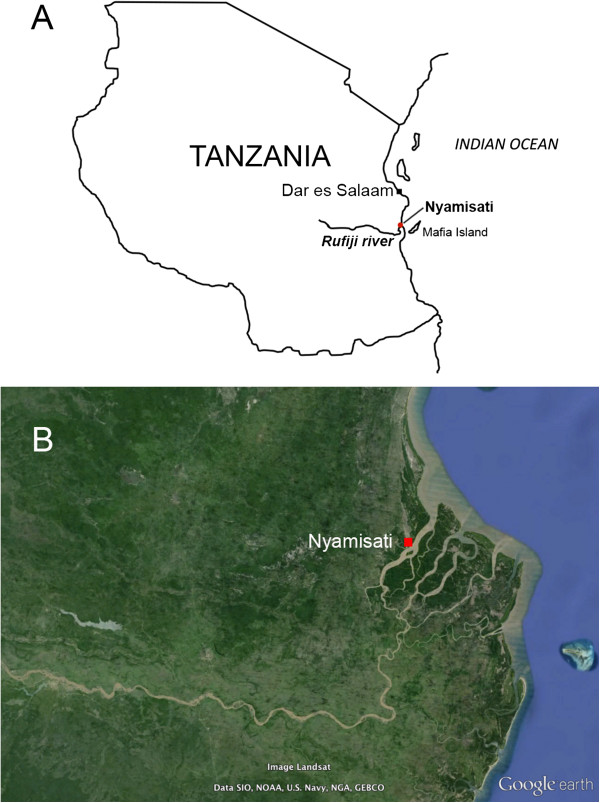
**Location of Nyamisati village, Tanzania A) Location of Nyamisati within the Rufiji District, Tanzania; B) Google Earth map showing Nyamisati in the Rufiji River Delta.**

Nyamisati village (7°47′S; 39°16′E) is situated in the Rufiji River Delta, the largest area of mangrove forest on the East African coast, in the Pwani region, Tanzania (Figure 1). The population is of Bantu origin and Muslim faith. Fishing and rice farming are the main occupations. Houses are mainly built in mud with roofs of grass and palm leaves. Malaria transmission is perennial with some seasonal fluctuations following the long and short rains, usually occurring in March-June and November-January, respectively (Additional file 1). HIV prevalence was 4.6% in adults in 1993. Sickle cell trait was detected by electrophoresis in 15% of villagers [3].

### Health care

A team, including a physician, a nurse and two locally trained assistants lived in the village 1984–2000. A health clinic was continuously available for consultations free of charge. Except for a few traditional healers, there were no other health facilities in this or neighbouring villages at the time. The health care management was gradually handed over to a governmental rural medical aid (RMA). Emergencies and more complicated medical conditions were treated by the physician or referred to Mchukwi Hospital, 72 km away.

Malaria was diagnosed by microscopy and treated with chloroquine during the first years and later on with sulphadoxine-pyrimethamine (SP), alone or in combination with oral quinine. The different anti-malarial drugs administered at the health unit are summarized in Additional file 2. Artemether-lumefantrine (Coartem®) was, according to the RMA, available in the village from 2009. Drugs were administered free of charge through the health unit. There were no other drug outlets in the village before 2000.

### Malaria control

In 1985, 3% of households owned a bed net. Between October 1993 and April 1994, 300 insecticide-treated bed nets (ITNs) were distributed to pregnant women and families with small children. The research team also distributed bed nets after the cross-sectional surveys in 1999 (900 ITNs not re-impregnated) and 2010 (1,000 long-lasting insecticide-treated nets (LLINs). The bed net coverage, assuming 1.8 individuals per net [22] was 4% in 1985, 45% in 1993–1994 and 100% after the survey in 1999. According to the villagers there was no other large-scale distribution of bed nets between 1999 and 2010. Indoor residual spraying and larval control were not used. Chemoprophylaxis with chloroquine and later intermittent preventive treatment (IPT) with SP were administered to pregnant women from a governmental maternal health unit. Except for a small study in 1986 [5], chemoprophylaxis and IPT were not administered to children.

### Malaria surveillance and cross-sectional surveys

A passive surveillance system for malaria was maintained between 1986–1989 and 1993–1999. The villagers were instructed to come to the clinic in the event of fever. Individuals who presented with fever (axillary temperature >37.5°C), or history of fever (“hot body”) within 24 hours, and had parasites detected by microscopy were administered anti-malarial treatment.

Cross-sectional surveys were performed in the population at the start of the long rainy season in 1986–1988, 1993–1999 and 2010. A subset of children aged ten to 19 years was sampled in January 2003 [11]. The cross-sectional surveys consisted of blood sampling and a general health assessment, including an axillary temperature and auscultation of heart and lungs. A spleen examination was performed in children and enlarged spleens were graded according to Hackett’s score [23]. Finger prick blood was collected for thick and thin films, Hemocue® blood haemoglobin testing (Ängelholm, Sweden), and optional HIV testing. Venous blood was collected in 4 ml EDTA tubes (for small children finger pricks in capillary tubes) and stored frozen as plasma and packed cells.

### Detection of malaria parasites by microscopy

Thick and thin films were stained with Giemsa and analysed by conventional light microscopy. Parasite densities were estimated per microlitre (μl) of blood and enumerated against the number of leukocytes assuming 8,000 leukocytes/μl. Blood films were considered negative if no parasites were detected in 100 microscopic fields of the thick smear.

### Detection of *Plasmodium falciparum*parasites by PCR

Parasite prevalence was assessed by PCR at the cross-sectional surveys 1994, 1995, 1999, and 2010 when most complete surveys across ages were performed. DNA was purified from frozen packed cells by Qiagen blood mini kit (Qiagen) (1994, 1995), phenol-chloroform extraction (1999), and using an automated Qiagen BioRobot® M48 (2010). Real-time PCR for detection of *Plasmodium* species was performed by a multiplex probe-based method [24] using the ABI TaqMan 7500, with a cut-off of 40 cycles to define positive samples. Genotyping of the *P. falciparum msp2* gene was performed by nested PCR using fluorescently labelled primers targeting the two allelic types of *msp2* in separate nested reactions, fragment sizing by capillary electrophoresis in a DNA sequencer (Applied Biosystems), and data analysis by GeneMapper software [25]. Data on *msp1* block 2 genotyping PCR from 2003 was available from a previous study [11]. The genotyping results are included here only with regard to detection of *P. falciparum*.

### Data analysis

Data analysis was performed using GraphPad Prism 5.0 (GraphPad Software Inc, USA) and Stata 13.0 (StataCorp, USA). The data from 1985–1988 were retrieved from summarized data [3, 4]. Parasite prevalence includes data from microscopy, *msp2*-PCR and real-time PCR. Missing prevalence data for *msp2*-PCR in 1986 and 1993, as well as for microscopy in 2010 (due to staining artefacts after storage), were estimated using the prevalence estimation tool developed by Okell *et al.*, validated for nested PCR data from all age cross-sectional surveys [26]. Agreement between methods was evaluated with the kappa statistic, using the Landis and Koch classification. Generalized estimating equation (GEE) regression models were used to account for statistical dependency of repeated observations when estimating the association between survey year and parasite prevalence, prevalence of sub-microscopic infections, and incidence of malaria. Complete population censuses were not performed; annual malaria incidence was therefore estimated among the individuals participating in the respective surveys. GEE logistic and negative binomial regression models were used to analyse binary (parasite prevalence), and count (malaria incidence) outcome variables. For each outcome, association with survey year was evaluated by univariate and multivariate models, adjusting for the effect of potential confounders and/or interactions (i.e., age, sex and fever and/or clinical episode of malaria at the time of survey. Anaemia was classified in four categories (non-anaemic, mild, moderate, and severe anaemia) based on haemoglobin levels according to WHO age and sex-specific criteria [27]. To evaluate changes in the overall prevalence of anaemia with time, a binary outcome defined as presence or absence of any level of anaemia was analysed using a univariate GEE logistic regression model. A univariate multinomial logistic regression model with robust clustering was used to evaluate changes in the prevalence of the four different levels of anaemia with time. Results from the multinomial logistic regression analysis are presented as predicted probabilities with 95% CI.

### Ethical considerations

All data and blood samples were collected with informed consent from each participant and/or their guardians. The project was approved by the Nyamisati Village Board, the Ethical Review Board of the National Institute for Medical Research in Tanzania and the Central Ethical Review Board in Stockholm, Sweden.

## Results

The population of Nyamisati increased from 451 individuals in 1985 to 1,553 individuals in 1999, as recorded by the research team. By 2012, 2,350 individuals lived in the village according to the National Bureau of Statistics in Tanzania [28]. The demographics of the individuals participating in the respective surveys are outlined in Table 1.

**Table 1:** **Characteristics of the study population at the respective cross-sectional surveys**

	1986	1993	1994	1995	1996	1997	1998	1999	2003	2010
**Population** ^**a**^ **, n**	500	1,125	1,295	1,396	1,458	1,424	1,507	1,553	n/a	n/a
**Cross-sectional survey, n**	470	555	792	712	326	337	509	889	105	808
**Female, n (%)**	239 (51.1)	300 (54.1)	448 (56.6)	403 (56.6)	205 (62.9)	187 (55.5)	276 (54.2)	476 (53.5)	42 (40.0)	407 (50.4)
**Age, y median (range)**	15 (0–90)	15 (0–83)	14 (0–84)	14 (0–84)	31 (1–86)	22 (1–82)	24 (2–78)	17 (1–84)	13 (10–19)	15 (1–82)
**Children ≤16 y, n (%)**	260 (55.3)	296 (53.3)	435 (54.9)	391 (54.9)	38 (11.7)	142 (42.1)	183 (36.0)	438 (49.3)	91 (86.7)	431 (53.3)
**Children 2–9 y, n (%)**	124 (26.4)	154 (27.7)	233 (29.4)	209 (29.4)	10 (3.1)	29 (8.6)	42 (8.2)	243 (27.3)	0	186 (23.0)
**Fever at survey, n (%)**	nd	6 (1.1)	78 (9.8)	89 (12.5)	28 (8.6)	5 (1.4)	25 (4.9)	152 (17.1)	1 (1.0)	23 (2.9)
**Fever at survey, 2–9 y, n (% of all febrile)**	nd	3 (50)	60 (77.9)	58 (65.2)	7 (25)	0	2 (8)	95 (62.5)	0	9 (39.1)

^a^The population size is based on the individuals registered in the research database at the respective years.

### Parasite prevalence by microscopy and PCR

**Figure 2:**
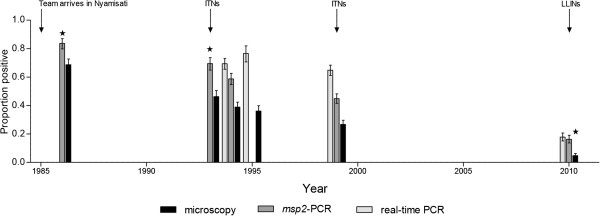
**Parasite prevalence in Nyamisati 1985–2010 including all ages, by microscopy and two PCR methods (** ***msp2*** **genotyping PCR and real-time species-specific PCR).** The parasite prevalence by *msp2-*PCR for 1986 and 1993 as well as microscopy for 2010 were estimated from slide and *msp2-*PCR data, respectively, using the prevalence estimation tool developed by Okell *et al*. [26] (as indicated by *).

The overall parasite prevalence by microscopy was 68.7% (95% CI 64.5-72.7%) in 1986–1988, 46.3% (95% CI 42.0-50.5%) in 1993, 38.9% (95% CI 35.5-42.4%) in 1994, and 26.7% (95% CI 23.8-29.7%) in 1999 (Figure 2). Species typing by microscopy defined ≥95% of parasite positive samples as *P. falciparum* in all surveys. In 2010, the microscopy prevalence was estimated at 4.8% (95% credible interval of prediction 3.6-6.3%) from the corresponding *msp2*-PCR data (according to [26]).

**Figure 3:**
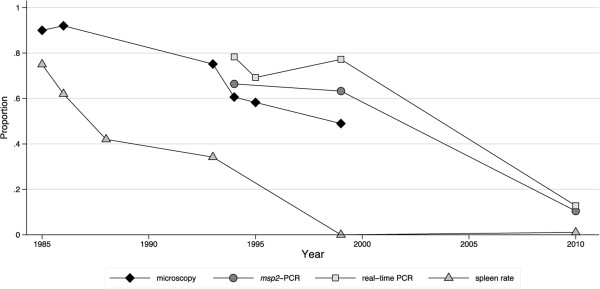
**Parasite prevalence and spleen rates in children aged two to nine years in repeated cross-sectional surveys in 1985–2010.** Parasite prevalence was assessed by microscopy, real-time PCR, and *msp2*-PCR. ITNs were distributed in October 1993-April 1994 (n = 300) and in 1999 after the survey (n = 900); LLINs were distributed after the survey 2010 (n = 1,000) (as indicated by arrows). The data from 1986–1988 are available only as published data, spleen rates are available for individual years, whereas parasite prevalence by microscopy is only available as pooled data.

**Figure 4:**
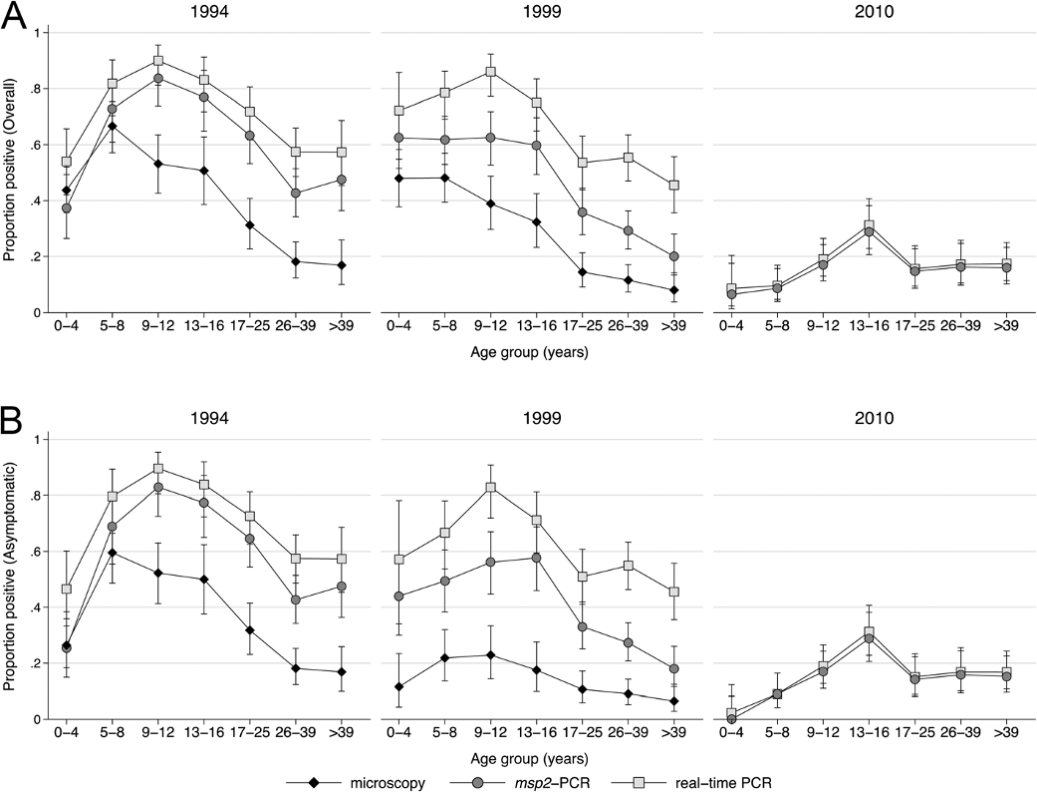
**Age patterns of parasite prevalence by microscopy, real-time PCR, and** ***msp2*** **-PCR A) overall and B) in asymptomatic individuals in 1994, 1999 and 2010.**

Both PCR methods consistently detected more *P. falciparum*-positive individuals than microscopy. The agreement between the real-time PCR and *msp2* PCR positivity was 80% whereas it was 57.4% with microscopy (Additional file 3). Real-time PCR detected higher parasite prevalence than *msp2-*PCR in all years, although the difference was less pronounced in 2010 (Figures 2, 3 and 4). The *msp2*-PCR prevalence in 1986, as estimated from microscopy prevalence in all ages by the method of Okell *et al*. [26], was 83.7% (95% credibility interval of prediction 79.8-87.0%). The *msp2-*PCR prevalence decreased in parallel with microscopy during the 1990s (1994 58.7%, 95% CI 54.7-62.2%; 1999 44.8%, 95% CI 41.5-48.2%), whereas real-time PCR positivity remained at similar levels (1994 69.4%, 95% CI 65.5-73.1%; 1999 64.8%, 95% CI 61.1-68.4%). In 2010, real-time PCR prevalence was 17.8% (95% CI 15.1-20.7%) and *msp2*-PCR 16.3% (95% CI 13.7-19.1%) (Figure 2).

### Asymptomatic parasite prevalence

The proportion of individuals who were febrile or had history of fever at the time of the cross-sectional survey varied over the years, being lowest in 1993 (1.1%) and highest in 1999 (17.0%) (Table 1). The asymptomatic parasite prevalence was lower than the overall prevalence in all surveys, with the largest difference among children aged 0–16 years in 1999 (Figure 4B). Among asymptomatic individuals, the odds of an infection being sub-microscopic increased significantly from 1994 to 1999 (GEE logistic regression age adjusted OR 1.73, 95% CI 1.20-2.49%).

### Parasite prevalence by age

In two to nine years old children, the age group conventionally used to estimate malaria endemicity [29], the parasite prevalence by microscopy was 90% in 1985, 75.2% in 1993, 60.6% in 1994, and 49.0% in 1999 (Figure 3). The prevalence by real-time PCR in this age group was 78.2% in 1994, 77.2% in 1999 and 12.8% in 2010 (Figure 3).

The peak parasite prevalence by microscopy gradually shifted to older children. In 1994–1999, the peak prevalence was seen in children aged five to eight years by microscopy and nine to 12 years by PCR. In 2010, the peak PCR prevalence had shifted to the 13–16 years group (Figure 4). The proportion sub-microscopic infections (negative by microscopy and positive by real-time PCR) increased with age. For instance, in 1999, 9.0% of asymptomatic individuals >16 years were parasite positive by microscopy and 51.3% by real-time PCR. The GEE logistic regression revealed that the odds of being parasite positive decreased with survey year in all age groups, with the most pronounced decrease in five to eight and nine to 12 years old children by real-time PCR (both OR 0.79, 95% CI 0.76-0.83%) (Table 2). In a subset of asymptomatic children in 2003, the parasite prevalence by microscopy was 3.1 and 13.6% in the ten to 12 and 13–16 years old children, respectively, and 46.9 and 46.6% by *msp1*-PCR [11].

**Table 2:** **Linear time-trend of odds of** ***Plasmodium falciparum*** **infection assessed by generalized estimating equation logistic regression models**

	All			Asymptomatic ^a^		
	OR (95% CI)			OR (95% CI)		
	Microscopy ^b^	***msp2*** -PCR ^c^	Real-time PCR ^d^	Microscopy ^b^	***msp2*** -PCR ^c^	Real-time PCR ^d^
**Year** ^**e**^						
**Age group**						
**0-4**	0.86 (0.83, 0.88)	0.90 (0.88, 0.91)	0.87 (0.85, 0.89)	0.73 (0.70, 0.77)	0.87 (0.86, 0.89)	0.85 (0.83, 0.86)
**5-8**	0.86 (0.83, 0.88)	0.80 (0.76, 0.85)	0.79 (0.76, 0.83)	0.73 (0.70, 0.77)	0.87 (0.86, 0.89)	0.85 (0.83, 0.86)
**9-12**	0.86 (0.83, 0.88)	0.82 (0.79, 0.86)	0.79 (0.76, 0.82)	0.73 (0.70, 0.77)	0.87 (0.86, 0.89)	0.85 (0.83, 0.86)
**13-16**	0.86 (0.83, 0.88)	0.90 (0.88, 0.91)	0.87 (0.85, 0.89)	0.73 (0.70, 0.77)	0.87 (0.86, 0.89)	0.85 (0.83, 0.86)
**>16**	0.86 (0.83, 0.88)	0.90 (0.88, 0.91)	0.87 (0.85, 0.89)	0.83 (0.78, 0.89)	0.87 (0.86, 0.89)	0.85 (0.83, 0.86)
**Age group** ^**f**^						
**0-4**	1 (-)	1 (-)	1 (-)	1 (-)	1 (-)	1 (-)
**5-8**	1.92 (1.46, 2.51)	3.20 (1.93, 5.31)	3.27 (1.97, 5.41)	2.60 (1.87, 3.61)	2.76 (1.70, 4.48)	2.39 (1.51, 3.77)
**9-12**	1.04 (0.79, 1.37)	3.87 (2.31, 6.48)	6.06 (3.53, 10.4)	1.86 (1.34, 2.59)	4.28 (2.67, 6.86)	4.73 (3.03, 7.40)
**13-16**	0.84 (0.63, 1.14)	2.14 (1.45, 3.16)	2.70 (1.82, 4.01)	1.65 (1.16, 2.36)	5.28 (3.26, 8.57)	4.92 (3.11, 7.77)
**>16**	0.23 (0.18, 0.30)	0.71 (0.52, 0.98)	1.04 (0.75, 1.45)	0.34 (0.25, 0.47)	1.56 (1.03, 2.36)	1.84 (1.25, 2.72)

^a^Asymptomatic defined as absence of fever, history of fever or treatment at survey.

^b^Based on microscopy data from 1993–1999.

^c^Based on real-time PCR data from 1994–2010.

^d^Based on *msp2*-PCR data from 1994–2010.

^e^Age-group specific effect of time (in years) on parasite prevalence.

^f^Effect of age on parasite prevalence at baseline.

In the GEE logistic regression analysis year of survey was treated as a continuous variable and age group as a categorical variable in five categories (ages: 0–4, 5–8, 9–12, 13–16, and >16 years). Due to significant interaction between year of survey and age group, effect of time on prevalence is presented as age group specific OR.

The odds of both overall and asymptomatic *P. falciparum* infection decreased with time and were most pronounced in asymptomatic individuals by microscopy.

*Abbreviations: OR* odds ratio, *CI* confidence interval, *msp2* merozoite surface protein 2 gene.

### Spleen rates

Enlarged spleens were recorded in >75% of two to nine years old children in 1985, 62% in 1986 and 42% in 1988 [3]. In 1993, spleen rate had decreased to 34.1% (95% CI 26.8-42.2%). During the following years, enlarged spleens were only sporadically recorded; and in 2010, 1.1% (95% CI 0.1-3.8%) of two to nine years old children had palpable spleens (Figure 3).

### Anaemia

**Figure 5:**
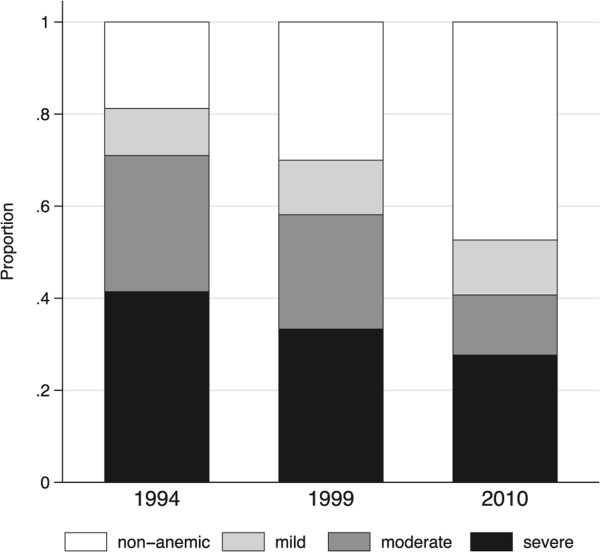
**Proportion of Nyamisati population with mild, moderate and severe anaemia at cross-sectional surveys, classified according to age and sex-specific WHO criteria** **[** [27] **]** **.**

According to the GEE logistic regression analysis, the proportion of individuals with any level of anaemia decreased significantly over time, and compared to 1994 the odds of anaemia was lower in 1999 (OR 0.61, 95% CI 0.47-0.79%) and further reduced in 2010 (OR 0.32, 95% CI 0.24-0.41%). Multinomial logistic regression analysis showed that the prevalence of severe and moderate anaemia decreased significantly from 41.4% (95% CI 36.4-46.5%) and 29.6% (95% CI 24.9-34.3%) in 1994 to 33.3% (95% CI 30.2-36.4%) and 24.8% (95% CI 22.0-27.7%) in 1999 and further to 27.6 (95% CI 24.5-30.7%) and 13.1% (95% CI 10.7-15.4%) in 2010, respectively. Prevalence of mild anaemia remained stable throughout the period at 10.2% (95% CI 7.1-13.3%), 11.9% (95% CI 9.7-14.0%) and 11.9% (95% CI 9.7-14.2%) in 1994, 1999 and 2010, respectively (Figure 5).

### Malaria morbidity and mortality

**Figure 6:**
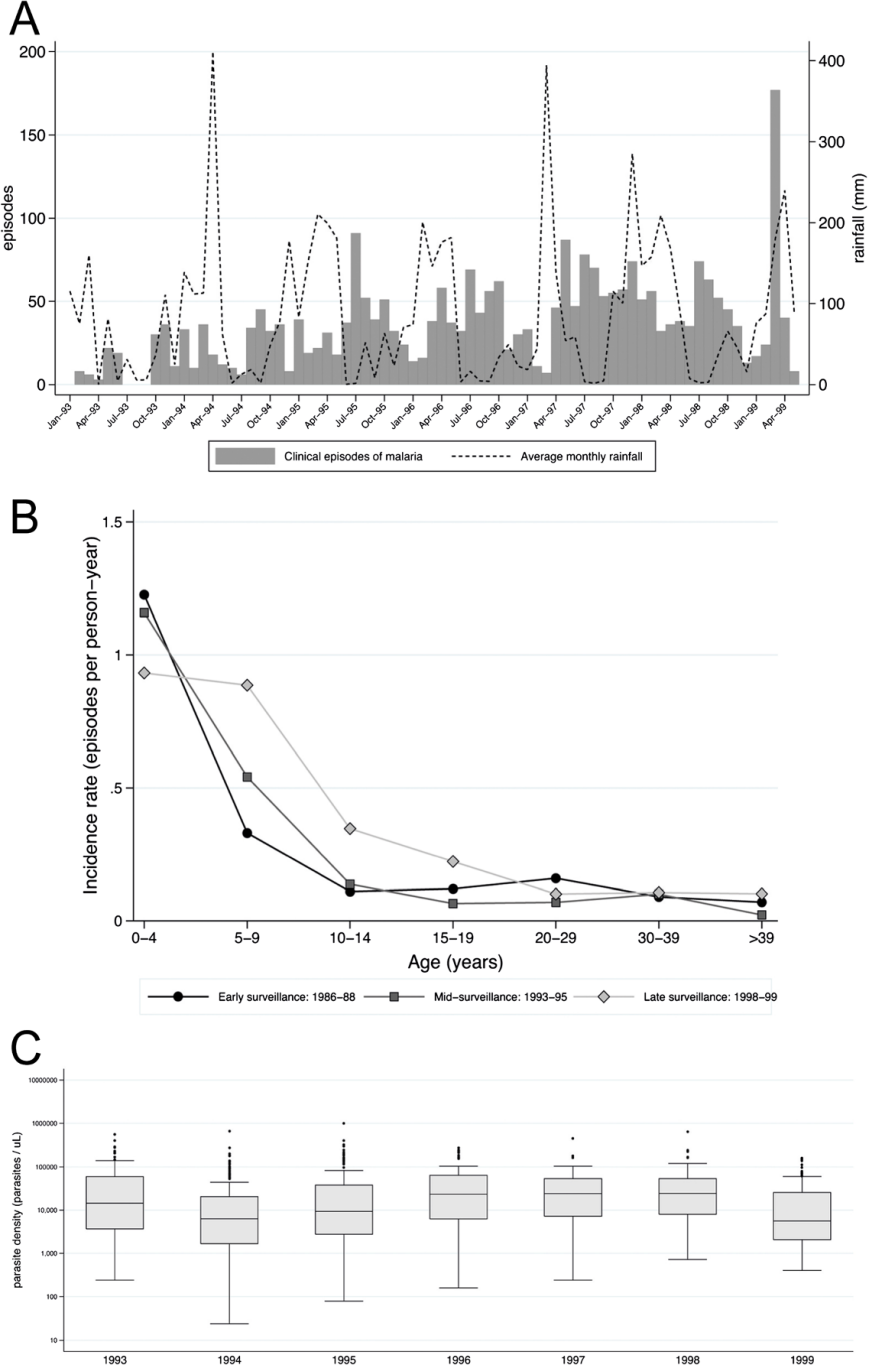
**Clinical episodes of malaria. A)** Number of clinical episodes of malaria diagnosed each month in 1993–1999 at the Nyamisati Health Clinic (bars) and rainfall patterns (solid line) averaged from data from the nearest meteorological stations of Utete, Kingupira, Kilwa Masoko, and Dar es Salaam International Airport, provided by the Tanzania Meteorological Agency; **B)** Incidence rate of clinical malaria by age over three observation periods 1986–1988, 1993–1995, and 1998–1999 (no data available 2010); **C)** Parasite densities in febrile malaria episodes over years.

Malaria episodes were diagnosed year round with some seasonal fluctuations, largely following rainfalls (Figure 6A). Data on malaria incidence was available from 1986–88 and 1993–1999. Since a systematic demographic surveillance of the village was not performed, the exact size of the population is unknown. Therefore, estimates of malaria incidence were restricted to the individuals that participated in the survey and known to be in the village during the period of surveillance. Malaria incidence was highest among the youngest children and decreased with increasing age in all years of surveillance (Figure 6B, Additional file 4). The incidence of malaria was similar within the respective age groups in 1986–1988 and 1993–1995, with an increase in the late 1990s. In 1999, a large portion of episodes coincided with the cross-sectional survey (Figure 6A).

Parasite densities >250,000 p/μl were detected in 1.7% of 515 febrile malaria episodes 1986–88 [3] and 0.6% of the 2,848 episodes in 1993–1999. Parasite densities in clinical malaria episodes varied between the years and increased from 1994 until 1998 (Figure 6C).

Severe anaemia was diagnosed in 1% of clinical malaria episodes in 1986–88 and 0.4% in 1993–1999. Seizures were reported in febrile children but there were no cases of unarousable coma. Other laboratory-based WHO criteria for severe malaria were not available. No deaths at the time of acute malaria were recorded when the research team lived in the village (1985–1991, 1993–2000).

## Discussion

The Nyamisati Malaria Research Project provides longitudinal data on the epidemiology of malaria in a rural African village. A substantial, yet gradual, decline from holo- to hypo-endemicity was observed over 25 years, as reflected by changes in parasite prevalence from 90 to <10% in two to nine years old children. Spleen rates decreased before changes in parasite prevalence were observed. Parasite prevalence by PCR, especially real-time PCR, remained high during the 1990s, and then revealed a substantial decrease by 2010.

This study adds to a number of reports of changing malaria epidemiology, giving hope for elimination of malaria in some settings [1, 19–21]. The long observation time contributes with historical data on malaria before the more recently introduced control interventions. Interestingly, very similar declines in malaria over the same time period have recently been reported from Muheza, Northern Tanzania [30] and Dielmo, Senegal [31].

The changes in transmission in Nyamisati were detected by different methods over time which demonstrates the advantage of using several metrics, i.e., spleen rates, microscopy and PCR in longitudinal surveillance. Molecular methods, especially PCR, have become increasingly used in epidemiological studies, and often reveal a large proportion of sub-microscopic infections [26]. The advantages and disadvantages of different methods to measure malaria transmission are well described in recent reviews (spleen rate not included) [32, 33].

The first malariometric index to capture changes in the burden of malaria was spleen rate, which decreased down to nil while parasite prevalence by microscopy in the same two to nine age group remained above 75%. Spleen rate (the proportion of children with enlarged spleens) was once the standard method to define malaria endemicity [29], but is no longer included in the WHO guidelines for malaria surveillance, being replaced by assessments of parasite prevalence [34]. Although spleen size is unspecific for malaria on the individual level, spleen rates may still be useful as a screening tool on the population level. The data suggest that assessment of spleen size in combination with microscopy can detect early changes in areas of most intense transmission, e.g. after implementation of new control interventions. This is supported by similar decreases in spleen rates, down to nil, observed as transmission declined in the longitudinal studies in north-eastern Tanzania [30] and Senegal [31].

Microscopy showed a gradual decrease in parasite prevalence over time. Among asymptomatic individuals, microscopy detected an even more rapid decrease in parasite prevalence, indicating the value of defining the clinical status of individuals in cross-sectional epidemiological surveys.

The use of PCR was found most informative when transmission had decreased to a moderate or low level, as suggested in previous reviews [32, 33]. PCR is well recognized to be a more sensitive method for parasite detection compared to microscopy, often revealing a large proportion of sub-microscopic infections in epidemiological studies [26]. This was also apparent here, especially as malaria declined in the 1990s. Furthermore, the choice of PCR method affects the interpretation of results on parasite prevalence. Including data from *msp2*-PCR allowed estimates of missing microscopy data according to the method by Okell *et al.,* which is based on nested PCR data [26]. Real-time PCR had higher sensitivity than *msp2*-PCR, likely due to differences in targeted genes (the multi-copy small subunit ribosomal RNA gene [35]*versus* the single copy *msp2* gene [36]). Real-time PCR prevalence remained high during the 1990s and would not alone have revealed the changes in transmission detected by the other methods. The high number of sub-microscopic infections detected by real-time PCR likely contributed to ongoing transmission in this setting [37], demonstrating the importance of using sensitive molecular methods to monitor epidemiology at stages of decreasing transmission.

The age patterns of parasite prevalence changed over the study period. The age group with the highest prevalence gradually shifted to older children, suggesting reduced immunity as a result of lower exposure as previously described [38, 39]. During the 1990s, increasing incidence of clinical malaria was observed which also might indicate a somewhat reduced immunity in the community as parasite prevalence declined during the 1990s. Moreover, a large number of symptomatic infections coincided with the cross-sectional survey in 1999, possibly reflecting changes to a more seasonal pattern of malaria transmission. The increasing incidence might, however, also reflect changes in drug susceptibility with increasing SP resistance. A similar trend was reported in Senegal before incidence eventually decreased in 2000s [31].

Cases of severe and fatal malaria were remarkably rare in the village. The continuous presence of a physician could have contributed to the paucity of severe malaria. However, malaria mortality was also low at the referral hospital in Mchukwi [40] and absence of malaria-specific mortality has been described in other settings of high transmission [31, 41]. Changes in transmission might have altered incidence and severity of malaria in the village as a result of lower exposure to parasites. Malaria case data was unfortunately not available after 2000. Nonetheless, the low parasite prevalence in 2010 reflects a level of unstable transmission with potential increased risk of malaria and severe malaria in all age groups [20, 33].

The proportion of individuals with severe and moderate anaemia declined over time. Increasing haemoglobin levels were also reported in the Pwani region in the Tanzania Demographic and Health Survey in 2010 [42]. There was no iron or vitamin supplementation programmes in Nyamisati. The decrease in parasite burden likely contributed to these changes since anaemia is an important consequence of symptomatic and asymptomatic *P. falciparum* infections [43].

Declining transmission has been associated with introduction of one or several interventions, such as ITN/LLIN and artemisinin-based combination therapy (ACT) [19, 31]. However, in some areas, such as in Uganda, malaria morbidity has increased despite scaling-up of control interventions [44]. The distribution of ITN in 1993–1994 and 1999 might partly explain the decreasing trends in parasite prevalence in Nyamisati, whereas ACT was not available until 2009 and access was low in the Rufiji district [45]. Nonetheless, decline in parasite burden as reflected by spleen rates and microscopy prevalence was observed already in the 1980s, i.e. before any of these control interventions were implemented. Also in other settings, such as The Gambia [21] and Kenya [20], declining transmission has been observed before the expansion of malaria control.

Interestingly, the declines in parasite prevalence over two decades in north-eastern Tanzania [30] and Senegal [31] are similar to the pattern observed in Nyamisati, despite differences regarding types and timing of interventions. In those areas, the *Anopheles* vector populations decreased markedly [46], along with changing vector species distribution [47] and entomological inoculation rates [31]. Entomological data are unfortunately not available in Nyamisati. In the Rufiji River Basin, the predominant species within the malaria-transmitting *Anopheles gambiae* complex were *Anopheles gambiae s.s, Anopheles arabiensis* and *Anopheles merus* in 2003–2004 [48]. Changes in rainfall or temperature might have impacted on the vector [49], nonetheless no apparent changing trends in these observations were registered in nearby weather stations in coastal Tanzania during this 25-year period (Additional file 1).

The presence of a research team closely monitoring malaria with prompt and efficacious treatment is likely to have contributed to reducing parasite prevalence in Nyamisati and other closely monitored communities [30, 31]. Already one year after the medical services were established in Nyamisati, mothers reported that their children’s health status had improved [3]. Nonetheless, the parasite prevalence continued to decrease after the research team left the village in 2000, suggesting that other factors contributed to the declining transmission. Also at a nearby Rufiji Demographic Surveillance Site (DSS), parasite prevalence decreased from 23 to 14% by microscopy during 2001–2006 [50]. Although the lack of data in Nyamisati between 2000 and 2010 is a limitation of the study, it is also interesting that transmission declined despite the research team not being present. A factor that could have contributed to the decline is improved socio-economic status [51]. The village was initially very remote and isolated. Transportation improved over the years with tarmac roads and daily busses that began coming to Nyamisati in 2004, as it became the main port for ferries to Mafia Island. Nevertheless, housing conditions in the village remained similar during the study period.

The study is limited by data not being equally available for all variables at all time points. Cross-sectional surveys only provide a snapshot of the malaria transmission and incidence data available for only part of the study period. Nonetheless, the major strength of the study is that the continuous malaria surveillance and cross-surveys were performed at the same time of the year (at the beginning of the rainy season) by the same physician and research team (including microscopy as well as clinical and spleen assessments), using the same sample collection tubes and processing, thus providing a unique consistency and comparability over this extended period of time. Moreover, only the survey years with complete data and samples available throughout the different ages were included in the analyses. Another limitation might be that DNA extractions were performed by different methods for the different years, which potentially could affect the sensitivity of detection. However, the PCR analyses were recently performed in a highly standardized fashion by the same individual. Moreover, the samples collected 1994 and 2010 were extracted by similar commercial kits (manual and automated, respectively) and can thus be considered highly comparable.

## Conclusion

The study demonstrates declining malaria transmission in this East African village over 25 years. Moreover it demonstrates the value of using different methods to monitor malaria transmission. Spleen rates and microscopy detect early changes when transmission is still intense. Conversely, PCR, especially real-time PCR, does not detect initial changes in transmission, but robustly and sensitively reveals a large proportion of sub-microscopic infections as transmission declines in a population.

The gradually declining transmission appears to have started already in the 1980s, with the arrival of the research team in 1985, and continued gradually after that. From the present data it is not possible to determine how much the interventions with ITNs contributed to the decline, partly because of the lack of data between 2000 and 2010. Further studies, using sero-epidemiological tools might contribute to the understanding of patterns and timing of transmission changes in periods when parasite prevalence data is missing. The historical data on malaria within this longitudinally, closely monitored rural village contributes to the understanding of changing epidemiology in sub-Saharan Africa over the last decades.

## Electronic supplementary material

Additional file 1: **Monthly rainfall (solid line) and temperature patterns, including high (dashed line) and low (dotted line) temperatures, in southeast coastal Tanzania from 1980 to 2010.** Data from meteorological stations of Utete, Kingupira, Kilwa Masoko, and Dar es Salaam International Airport, kindly provided by Tanzania Meteorological Agency. (PDF 90 KB)

Additional file 2: **Type of anti-malarial drugs administered through the health and research unit during the 1990s.** (PDF 867 KB)

Additional file 3: **Agreement between real-time PCR results with microscopy and** ***msp2*** **-PCR.** (DOCX 11 KB)

Additional file 4: **Malaria incidence in Nyamisati 1993–1999 evaluated using univariate and multivariate generalized estimating equation negative binomial regression models.** (DOCX 13 KB)

## Abbreviations

EDTA: Ethylenediamine tetra acetic acid
GEE: Generalized estimating equation
HIV: Human immunodeficiency virus
IPT: Intermittent preventive treatment
ITN: Insecticide-treated nets
LLIN: Long-lasting insecticide-treated nets
*msp1*: Merozoite surface protein 1 gene
*msp2*: Merozoite surface protein 2 gene
PCR: Polymerase chain reaction
RMA: Rural medical aid
SP: Sulphadoxine-pyrimethamine.
