# Supplementary figures and images for: Epidemiology of malaria in a village in the Rufiji River Delta, Tanzania: declining transmission over 25 years revealed by different parasitological metrics
Source: Malar J. 2014 Nov 26;13:459. doi: 10.1186/1475-2875-13-459 (PMC4289390; doi:10.1186/1475-2875-13-459)

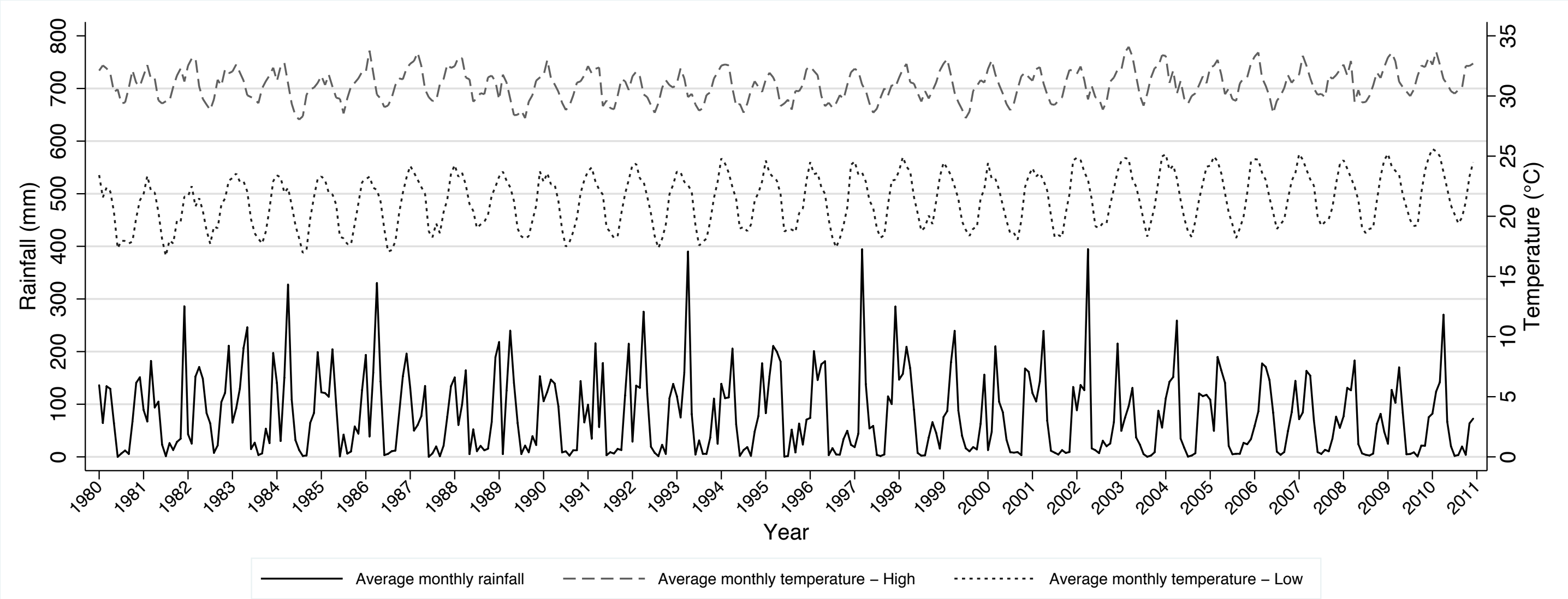

Supplement: Supplementary file 1 — Additional file 1: Monthly rainfall (solid line) and temperature patterns, including high (dashed line) and low (dotted line) temperatures, in southeast coastal Tanzania from 1980 to 2010. Data from meteorological stations of Utete, Kingupira, Kilwa Masoko, and Dar es Salaam International Airport, kindly provided by Tanzania Meteorological Agency. (PDF 90 KB) [file 12936_2014_3649_MOESM1_ESM.pdf]

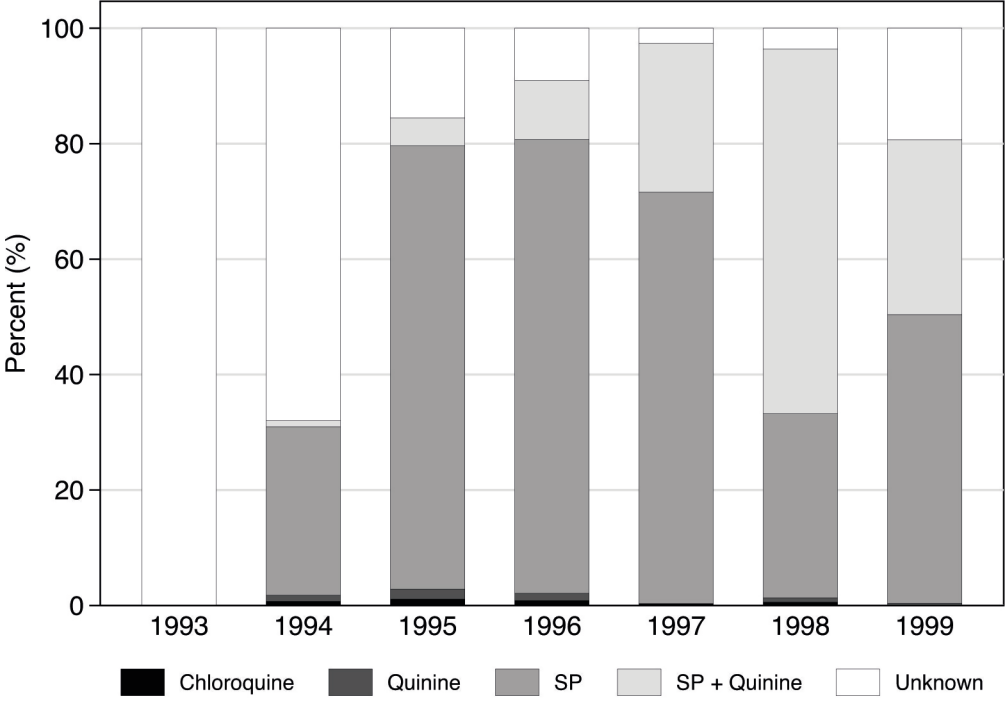

Supplement: Supplementary file 2 — Additional file 2: Type of anti-malarial drugs administered through the health and research unit during the 1990s. (PDF 867 KB) [file 12936_2014_3649_MOESM2_ESM.pdf]
