# Supplementary material for: Epidemiology of malaria in a village in the Rufiji River Delta, Tanzania: declining transmission over 25 years revealed by different parasitological metrics
Source: Malar J. 2014 Nov 26;13:459. doi: 10.1186/1475-2875-13-459 (PMC4289390; doi:10.1186/1475-2875-13-459)
Supplement: Supplementary file 3 — Additional file 3: Agreement between real-time PCR results with microscopy and msp2 -PCR. (DOCX 11 KB) [file 12936_2014_3649_MOESM3_ESM.docx]

**Additional file 3. Agreement between real-time PCR results with microscopy and *msp2*-PCR**

| **Agreement between real-time PCR and other methods** | | | | | | | |
| --- | --- | --- | --- | --- | --- | --- | --- |
| **Method** | **Agreement**  **%** | **PPA^a^**  **%** | **PNA^b^**  **%** | **Kappa** | **Std. Err.** | **Z** | **P** |
| **Microscopy** | 57.4 | 41.2 | 39.3 | 0.239 | 0.020 | 11.78 | <0.001 |
| ***msp2*-PCR** | 80.0 | 70.6 | 61.7 | 0.604 | 0.026 | 23.07 | <0.001 |

^a^ Percent positive agreement

^b^ Percent negative agreement
