# Supplementary material for: Epidemiology of malaria in a village in the Rufiji River Delta, Tanzania: declining transmission over 25 years revealed by different parasitological metrics
Source: Malar J. 2014 Nov 26;13:459. doi: 10.1186/1475-2875-13-459 (PMC4289390; doi:10.1186/1475-2875-13-459)
Supplement: Supplementary file 4 — Additional file 4: Malaria incidence in Nyamisati 1993–1999 evaluated using univariate and multivariate generalized estimating equation negative binomial regression models. (DOCX 13 KB) [file 12936_2014_3649_MOESM4_ESM.docx]

**Additional file 4. Malaria incidence in Nyamisati 1993-1999** evaluated using univariate and multivariate GEE negative binomial regression models. The multivariate analysis takes the different age distribution at surveys into account.

|  | **Univariate^a^** | | **Multivariate^b^** | |
| --- | --- | --- | --- | --- |
| **Year** | **IRR (95% CI)** | ***P*** | **IRR (95% CI)** | ***P*** |
| **1993** | 0.69 (0.56, 0.85) | <0.001 | 0.65 (0.50, 0.85) | 0.002 |
| **1994^c^** | 1.00 (ref) | - | 1.00 (ref) | - |
| **1995** | 1.75 (1.49, 2.05) | <0.001 | 1.86 (1.52, 2.28) | <0.001 |
| **1996** | 1.13 (0.91, 1.42) | 0.273 | 2.39 (1.70, 3.36) | <0.001 |
| **1997** | 1.07 (0.85, 1.33) | 0.570 | 2.06 (1.52, 2.80) | <0.001 |
| **1998** | 1.05 (0.87, 1.28) | 0.593 | 2.21 (1.69, 2.89) | <0.001 |
| **1999** | 1.21 (1.02, 1.42) | 0.026 | 1.86 (1.52, 2.29) | <0.001 |
| **Age** |  |  |  |  |
| **1-4** |  |  | 1.00 (ref) | - |
| **5-8** |  |  | 0.56 (0.46, 0.67) | <0.001 |
| **9-12** |  |  | 0.22 (0.17, 0.28) | <0.001 |
| **13-16** |  |  | 0.14 (0.11, 0.19) | <0.001 |
| **17-25** |  |  | 0.08 (0.06, 0.10) | <0.001 |
| **26-39** |  |  | 0.07 (0.05, 0.09) | <0.001 |
| **40-** |  |  | 0.04 (0.03, 0.07) | <0.001 |

^a^ Results from univariate GEE negative binomial regression model

^b^ Results from multivariate GEE negative binomial regression model, adjusting for age (in seven categories: ages <5, 5-8, 9-12, 13-16, 17-25, 26-39, >39 years).

^c^ Since 1993 appears to an unusual year with regards to number of episodes (Figure 6A), the annual incidence rate ratios during the 1990s were compared to 1994.

Abbreviations: IRR, incidence rate ratio; CI, confidence interval
